# School guidance support and growth in mathematics discourse feedback skills: a three-wave longitudinal mediation study

**DOI:** 10.3389/fpsyg.2025.1725689

**Published:** 2026-01-07

**Authors:** Yekun Liu, Mohong Wu, Juncheng Guo, Xiaocun Huang

**Affiliations:** 1 School of Physics and Information Engineering, Cangzhou Normal University, Cangzhou, Hebei, China; 2 Union Committee, Cangzhou Normal University, Cangzhou, Hebei, China; 3 Faculty of Social Sciences and Liberal Arts, UCSI University, Kuala Lumpur, Malaysia

**Keywords:** feedback literacy behavior, longitudinal structural equation modeling, major decision-making self-efficacy, mathematics discourse feedback skills, school guidance support, secondary education, sequential mediation

## Abstract

**Introduction:**

Feedback improves learning when students can interpret evaluative information, justify reasoning, monitor progress against explicit criteria, diagnose errors, and implement targeted revisions in classroom dialogue. This study tested whether school guidance support fosters growth in mathematics discourse feedback skills through motivational and behavioral mechanisms.

**Methods:**

We used a three-wave longitudinal design with Chinese senior high school students. School guidance support was assessed at T1, major decision-making self-efficacy at T2, feedback literacy behavior at T3, and mathematics discourse feedback skills at all waves. Longitudinal measurement invariance was established for the outcome. Hypotheses were evaluated with structural equation modeling that controlled for prior levels of the outcome and demographic covariates. Missing data were handled with full-information maximum likelihood, and indirect effects were tested with bias-corrected bootstrapping.

**Results:**

School guidance support predicted later mathematics discourse feedback skills both directly and indirectly. Indirect effects emerged *via* T2 major decision-making self-efficacy and *via* T3 feedback literacy behavior. A sequential pathway from T1 guidance support to T2 self-efficacy to T3 feedback literacy behavior, and in turn to T3 mathematics discourse feedback skills, was also supported. The pattern is consistent with a time-ordered mechanism linking contextual supports, agentic beliefs, feedback-using behaviors, and discipline-specific skills.

**Discussion:**

Findings integrate social cognitive, feedback literacy, and self-regulated learning perspectives by specifying how school-level supports translate into domain-specific competencies. Practically, the results suggest a design principle for secondary schools: strengthen decision-making self-efficacy early, engineer routine opportunities to seek, interpret, and apply feedback, and then assess discourse-based reasoning and revision as proximal indicators of skill growth.

## 1 Introduction

Feedback improves learning when students effectively engage in mathematics discourse feedback skills, defined as the capacity to interpret evaluative information, justify reasoning, monitor progress against explicit criteria, diagnose errors, and implement targeted revisions in classroom dialogue (Heron et al., 2023; Koichu, 2019). In mathematics, these discourse-oriented practices are essential for converting feedback into improvement and sustaining participation in demanding discussions (Webb et al., 2019). Many secondary students, however, find it difficult to turn comments into action when tasks are complex or expectations are not transparent, which keeps the field focused on the conditions under which feedback is used rather than merely delivered (Ketonen et al., 2020).

Two complementary levers are central in this problem space. At the school level, guidance services can offer informational clarity, credible modeling of effective decision processes, and structured opportunities to seek formative input (Raynham et al., 2023). At the learner level, motivational beliefs and feedback literacy behaviors, specifically the active actions of seeking, making sense of, and utilizing feedback information (Carless and Boud, 2018), shape whether available feedback is effectively applied (Gao and Brown, 2023).

Building on social cognitive perspectives, feedback literacy accounts, and self-regulated learning in dialogic classrooms, we consider a time-ordered process in which school guidance support strengthens students’ major decision-making self-efficacy (i.e., confidence in gathering information and making academic choices), which then facilitates feedback literacy behavior, culminating in stronger mathematics discourse feedback skills. Addressing these processes is urgent given the persistent challenge of “feedback waste,” where valuable evaluative information is often ignored by students due to a lack of agency or strategic know-how (Pitt and Norton, 2017). While recent scholarship has pivoted from focusing solely on teacher delivery to emphasizing student feedback literacy (Little et al., 2024; Nieminen and Carless, 2023), empirical research linking these general regulatory behaviors to domain-specific enactments, such as mathematics discourse skills, remains scarce. Understanding this translation is critical for effective instruction, as it clarifies how general student competencies function as scaffolds for disciplinary performance (Lee et al., 2020).

The present study contributes to this emerging literature with three layers of novelty. First, unlike prior research that typically treats feedback literacy and disciplinary skills in isolation, we interrogate the developmental mechanism through which general feedback literacy behaviors translate into specific mathematics discourse feedback skills (Webb et al., 2019). Second, we bridge the often-siloed fields of school counseling and subject-matter instruction by testing the novel proposition that macro-level guidance acts as an essential antecedent for micro-level classroom dialogue (Taylor et al., 2017). Finally, by employing a three-wave longitudinal design, we move beyond cross-sectional snapshots to capture the temporal ordering of these constructs, responding to recent calls for rigorous longitudinal evidence in feedback research.

### 1.1 School guidance support and mathematics discourse feedback skills

Mathematics discourse feedback skills refer to students’ capacity to interpret evaluative information, articulate and justify mathematical reasoning, monitor progress, diagnose errors, and implement revisions in classroom dialogue (Zhang et al., 2025). These skills matter because they translate feedback into purposeful action and sustain participation in rigorous mathematical discussion, which is central to achievement in secondary education (Xu et al., 2021). Consistent with this view, research in mathematics classrooms indicates that students who can explain and justify their solutions, diagnose errors, and revise their work in response to feedback tend to obtain higher scores on curriculum-aligned assessments and display more adaptive achievement trajectories (Smit et al., 2023b). The High School Students’ Mathematics Discourse Feedback Skills Scale (MDFSS; Chen et al., 2024) was developed to capture this constellation of discourse-based feedback skills in Chinese senior high schools, and initial validation work has shown that the measure behaves as expected in relation to other indicators of mathematics learning. At the same time, systematic evidence directly linking MDFSS scores to objective academic performance, such as standardized test scores or course grades, remains limited. The present study therefore treats mathematics discourse feedback skills as a proximal, theoretically grounded outcome whose precise associations with distal achievement indicators require further investigation.

A theoretically grounded pathway links school guidance support to these skills. Social cognitive theory proposes that structured environmental supports shape efficacy beliefs and outcome expectations through informational resources, modeling, and persuasive feedback, which in turn promote strategic engagement and persistence when facing challenge (Fort and Cerqueira, 2025; Riley et al., 2015). Cyclical models of self-regulated learning explain how clarity of goals and standards facilitates planning, how guided monitoring strengthens adaptive control during performance, and how supported reflection consolidates learning, all of which align with interpreting and applying feedback in mathematics (Callan et al., 2021; Callan and Cleary, 2019). Sociocultural and dialogic perspectives further hold that participation in disciplinary discourse depends on access to shared tools, common language, and explicit norms for explanation and critique (de Lange and Wittek, 2022; Mercer and Howe, 2012). School guidance support can provide clarity about performance expectations, organize opportunities for feedback-rich interaction with teachers and peers, and cultivate a climate that values explanation, error diagnosis, and iterative improvement (Keiler et al., 2020). These conditions increase both the frequency and quality of feedback encounters and foster more advanced discourse-related skills. Accordingly, we propose the following hypothesis:

*H1:* School guidance support is positively associated with students’ mathematics discourse feedback skills.

### 1.2 Major decision-making self-efficacy as a mediator

Major decision-making self-efficacy is the belief that one can gather and evaluate information, make academically significant choices, and sustain those choices under uncertainty (Yang and Delgado, 2025). Social cognitive theory posits that efficacy develops through mastery experiences, vicarious experiences, verbal persuasion, and the regulation of physiological and affective states, all of which can be cultivated by well-structured school guidance (Capa-Aydin et al., 2018). Social cognitive career theory further proposes that contextual supports shape educational behaviors primarily by strengthening efficacy beliefs (Wang et al., 2022). When guidance provides credible information about options, visible models of sound decision-making processes, timely encouragement, and opportunities to rehearse choices in low-risk settings, students are more likely to appraise themselves as capable decision makers (Bandura, 2023; Niu et al., 2025).

Higher decision-making self-efficacy should translate into mathematics discourse feedback skills in class. Students who feel capable set clearer goals, adopt adaptive strategies, and persist through difficulty, which increases the likelihood of approach-oriented participation in feedback-rich exchanges (Ren et al., 2025; Stephen et al., 2020). Such students more readily seek clarifying input, articulate and justify mathematical reasoning, monitor progress against standards, and implement revisions that reduce performance–standard discrepancies (Labuhn et al., 2010). Repeated engagement in these behaviors consolidates into more advanced discourse-related competencies that are visible in mathematical explanation, diagnostic evaluation, and effective implementation (Erath et al., 2018; Xu et al., 2023). We, therefore, propose the following hypothesis:

*H2:* Major decision-making self-efficacy mediates the association between school guidance support and mathematics discourse feedback skills.

### 1.3 Feedback literacy behaviors as a mediator

Feedback literacy behaviors are the enacted capacities to appreciate the purposes of feedback, make informed judgments about quality, regulate affect during appraisal, and take action to improve subsequent work (Dawson et al., 2024). The feedback literacy framework treats these capacities as learnable and socially situated, developing when learners encounter clear standards, exemplars, and structured opportunities to discuss evidence and rehearse revision in supportive settings (Curtis et al., 2025; de Kleijn, 2023).

First, school guidance support is expected to strengthen feedback literacy behaviors. Within the feedback literacy framework, clarity of expectations, access to models of quality, and opportunities for dialogic appraisal are the key affordances that convert evaluative information into action (Chinpakdee, 2025; Jin et al., 2024). Guidance services provide precisely these affordances by setting explicit performance criteria, organizing exposure to exemplars, arranging teacher and peer conferencing, and normalizing feedback-seeking and resubmission (Winstone et al., 2017). These conditions lower the social and emotional cost of seeking feedback and create repeated practice in interpreting and using evaluative information, thereby consolidating feedback literacy as a habitual repertoire (Joughin et al., 2021).

Second, feedback literacy behaviors are expected to advance mathematics discourse feedback skills. Self-regulated learning theory explains how effective learning unfolds across forethought, performance, and reflection (Khiat and Vogel, 2022). Students who routinely seek and make sense of feedback enter tasks with clearer goals, monitor problem-solving more accurately, and engage in targeted revision afterward (Bouwer and Dirkx, 2023). In mathematics discourse, these regulatory routines manifest as willingness to articulate and justify reasoning, to request clarification when uncertainty arises, to diagnose errors with precision, and to implement revisions that align work with task criteria (Seah and Horne, 2020). Through repeated feedback-rich exchanges, these behaviors accumulate into stronger discourse-related competencies (Carless and Young, 2024). Together, these theoretical accounts imply a process in which school guidance support fosters feedback literacy behaviors, which, in turn, enhance mathematics discourse feedback skills. We, therefore, propose the following hypothesis:

*H3:* Feedback literacy behaviors mediate the association between school guidance support and mathematics discourse feedback skills.

### 1.4 Sequential mediation via major decision-making self-efficacy and feedback literacy behaviors

School guidance support provides informational clarity, credible models, and structured opportunities to rehearse academically significant choices (Corwin et al., 2004). In social cognitive theory, such conditions are the classic sources that cultivate self-efficacy, while social cognitive career theory specifies that school-level supports shape educational behaviors largely through their effects on efficacy beliefs (Demanet and Van Houtte, 2024; Zhang et al., 2021). When students appraise themselves as capable decision makers, they are more likely to adopt approach-oriented goals, to persist under uncertainty, and to deploy adaptive strategies in evaluative learning situations (Schweder et al., 2025).

Greater major decision-making self-efficacy is then expected to prime the enactment of feedback literacy behaviors. The feedback literacy framework holds that productive uptake of evaluative information requires appreciating the purposes of feedback, making judgments about quality, managing affect, and taking action (Carless and Winstone, 2023). Efficacy-confident students are more willing to engage in feedback-seeking, to expose interim reasoning to scrutiny, and to translate comments into targeted revisions (Wei et al., 2024). Self-regulated learning theory explains why this translation occurs, as clearer forethought goals, more accurate monitoring during performance, and reflective adaptation after performance align students’ regulation with task criteria (Guo et al., 2025; Raković et al., 2022).

As these behaviors become habitual, they are expressed in mathematics discourse as clearer explanation, justified argumentation, calibrated monitoring of progress, precise diagnosis of errors, and efficient implementation of improvements (Biza et al., 2018). In other words, feedback-literate participation provides the immediate practices through which a supportive school context and a strong sense of decision-making capability are realized as discipline-specific discourse-related skills (Yu and Liu, 2021). Accordingly, we propose the following hypothesis:

*H4:* Major decision-making self-efficacy and feedback literacy behaviors sequentially mediate the association between school guidance support and mathematics discourse feedback skills.

### 1.5 The current study

**Figure 1:**
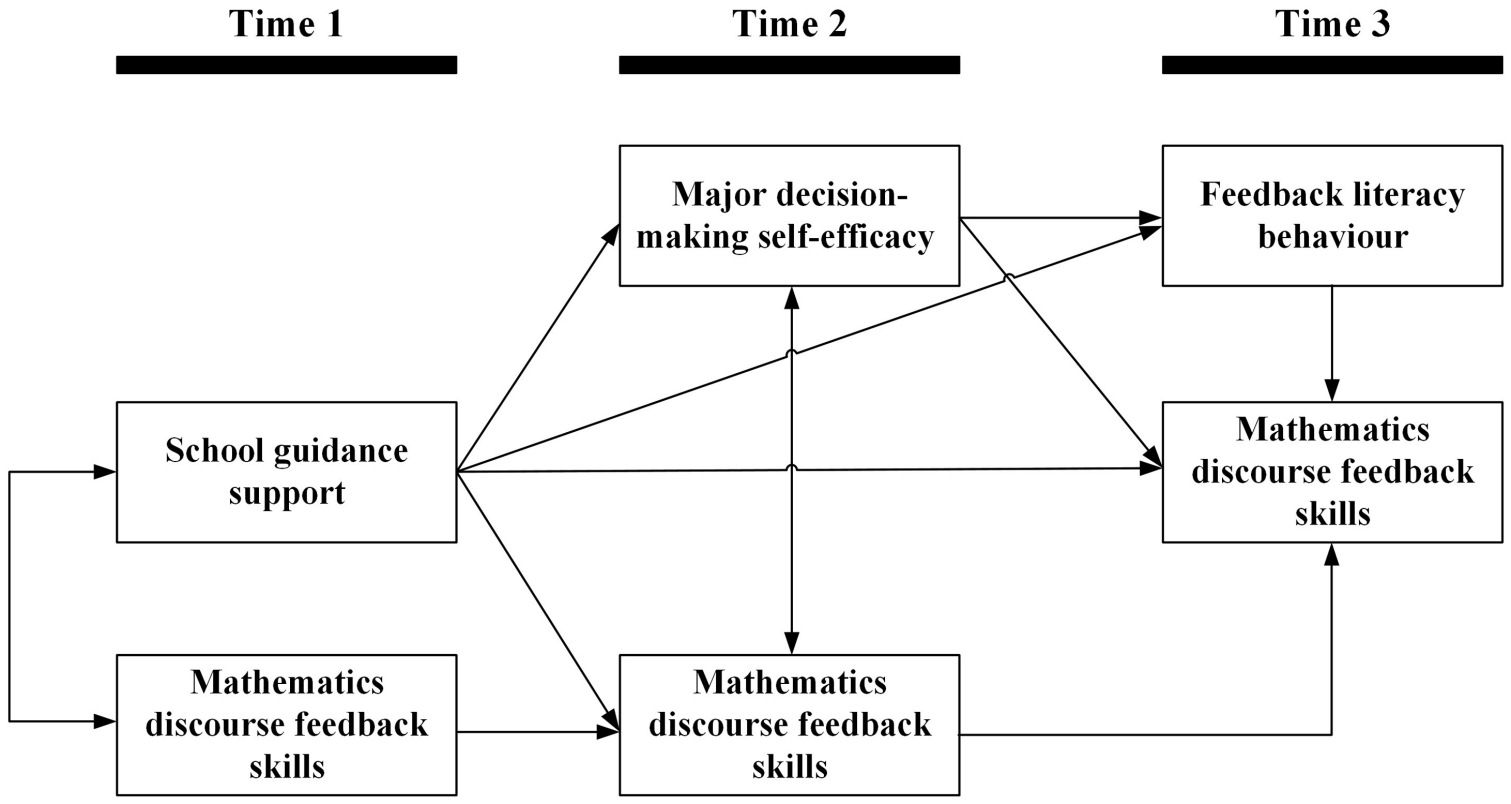
Hypothesized three-wave longitudinal mediation model linking student perceptions of school guidance support (T1), major decision-making self-efficacy (T2), feedback literacy behavior (T3), and mathematics discourse feedback skills (T1–T3).

Guided by the framework described above, the present study employed a three-wave longitudinal design with approximately 3-month intervals to examine the processes depicted in Figure 1. We utilized a sample of Chinese high school students to investigate the developmental mechanisms linking contextual support to domain-specific skills. Specifically, we assessed student perceptions of school guidance support at Time 1, major decision-making self-efficacy at Time 2, feedback literacy behavior at Time 3, and mathematics discourse feedback skills across all three waves. To isolate the unique effects of the hypothesized predictors, we controlled for adolescent age, gender, grade level, place of origin, and parental education. Based on the theoretical integration of social cognitive and feedback literacy perspectives, we tested four specific hypotheses:

- *H1:* Student perceptions of school guidance support are positively associated with subsequent mathematics discourse feedback skills.
- *H2:* Major decision-making self-efficacy mediates the association between school guidance support and later mathematics discourse feedback skills.
- *H3:* Feedback literacy behavior mediates the association between school guidance support and later mathematics discourse feedback skills.
- *H4:* Major decision-making self-efficacy and feedback literacy behavior sequentially mediate the association between school guidance support and later mathematics discourse feedback skills.

## 2 Method

### 2.1 Participants and procedure

Using convenience sampling, we recruited 2,120 adolescents from three senior high schools in Hebei, China. At Time 1 (T1; March 2025), participants were 15–18 years old (*M*_age_ = 16.47, SD = 0.97), including 1,055 boys and 1,065 girls. By grade, 762 students were in Grade 10, 660 in Grade 11, and 698 in Grade 12. Regarding place of residence, 79.7% were from urban areas and 20.3% were from rural areas. For parents’ highest educational attainment, 42.5% reported a bachelor’s degree, 34.9% reported an associate degree or below, and 22.6% reported a graduate degree or above. Each school served as an independent sampling frame defined by intact classes in Grades 10–12 that were scheduled during the class period used for data collection. Counselors first briefed all eligible classes and invited all students present to participate with parental consent. At School 1, 720 eligible students were approached and 690 consented and completed T1 (response rate = 95.8%). At School 2, 730 were approached and 707 completed T1 (response rate = 96.8%). At School 3, 770 were approached and 723 completed T1 (response rate = 93.9%). The overall school-level response rate at T1 was 95.5%.

Inclusion criteria were full-time enrollment in Grades 10–12 at one of the participating schools, age within 15–18 years, sufficient literacy to complete a self-report questionnaire in Chinese, and provision of adolescent assent and parental consent. There were no exclusions based on academic achievement or disability status. To ensure data quality, questionnaires contained two instructed-response items. We also computed long-string indices within multi-item scales and inspected item-level variability to identify patterned responding. A case was excluded at a given wave if it failed both instructed-response items or displayed a long-string index indicating the same response for more than 90% of items on a scale in combination with negligible variance across the remaining items. The Ns reported for each wave reflect data after applying these screening criteria. All instruments were administered in Mandarin Chinese. For measures adapted from English, bilingual psychologists used translation and back-translation procedures, followed by cognitive interviews with a small group of students to confirm clarity and cultural appropriateness.

To match responses longitudinally while preserving anonymity, each participant received at T1 a preprinted card bearing a random alphanumeric code. The same code was copied by the student onto the questionnaire at each wave, and the list of codes contained no names or contact information. Codes were used solely for matching across waves and were stored separately from the analytic data. If a student lost a code card, a new code was issued, and the case was treated as unmatched for longitudinal linking.

Three months later (T2; June 2025), the same procedures were followed and 2,032 students participated, representing 95.8% retention from T1 (1,009 boys and 1,023 girls). After a further 3 months (T3; September 2025), 1,938 students remained in the study (958 boys and 980 girls). The overall attrition rate from T1 to T3 was 8.6%. Attrition at each wave was primarily due to student absence on the day of data collection, most often because of illness or school-arranged activities. During the longitudinal period, all participants continued to attend their original schools.

Ethical approval was granted by the ethics committee of the corresponding author’s institution. Before data collection, we obtained both oral and written informed consent from all adolescent participants, as well as signed parental consent for each. Students were informed that participation was voluntary and that they could withdraw at any time without penalty. Trained counselors administered the surveys in classrooms during regular school hours following a standardized protocol, which was repeated at all three waves. No incentives were provided.

Missing data arising from attrition or occasional item non-response were addressed in the structural equation models using the estimation approach described in the Analytic Strategies section (Section 2.3). For constructs measured repeatedly, longitudinal measurement invariance across T1–T3 was evaluated in Mplus by fitting configural, metric, and scalar models prior to estimating the substantive longitudinal mediation model.

### 2.2 Measures

#### 2.2.1 Student perceptions of school guidance support (T1)

Student perceptions of school guidance support were assessed at T1 using the Student Perceptions of School Guidance Support Scale (Xu and Zhu, 2024). The instrument comprises 32 items divided into four domains: academic development, career development, personal-social development, and college preparation. A sample item is “My school helps me discover my personal interests.” Items are rated on a 5-point Likert-type scale ranging from 1 (strongly disagree) to 5 (strongly agree), with higher scores indicating stronger perceived guidance support. Prior validation with Chinese senior high school students demonstrated satisfactory construct validity *via* confirmatory factor analysis, and it supported the use of an overall composite score (Xu and Zhu, 2024). In the present study, internal consistency reliability was high, with a coefficient omega (*ω*) of 0.91.

#### 2.2.2 Major decision-making self-efficacy (T2)

Major decision-making self-efficacy was assessed at T2 using the Major Decision-Making Self-Efficacy Scale (Li et al., 2025), developed specifically for the Chinese high school context. The scale consists of 24 items assessing five domains: information acquisition, decision persistence, self-determination, self-evaluation, and social support. A sample item is “I am confident that I can find information about the majors I am interested in.” Responses are given on a 5-point Likert-type scale from 1 (strongly disagree) to 5 (strongly agree). The original validation study reported a clear five-factor structure, high composite reliability, and evidence of convergent validity (Li et al., 2025). In the current study, the scale demonstrated good internal consistency (*ω* = 0.85).

#### 2.2.3 Feedback literacy behavior (T3)

Feedback literacy behavior was measured at T3 using the Chinese version of the Feedback Literacy Behavior Scale (FLBS; Dawson et al., 2024). The instrument includes 24 items capturing five behavioral domains: seeking feedback information, making sense of information, using feedback information, providing feedback information, and managing affect. A sample item is “I seek out examples of good work to improve my work.” Items are rated on a 6-point Likert-type scale from 1 (never) to 6 (always). Previous validation with Chinese learners confirmed the five-factor structure and established measurement invariance across gender and education sectors (Zhu et al., 2025). In this study, the scale showed excellent internal consistency (*ω* = 0.93).

#### 2.2.4 Mathematics discourse feedback skills (T1, T2, T3)

Mathematics discourse feedback skills were assessed at all three waves using the High School Students’ MDFSS (Chen et al., 2024). This 24-item instrument measures six dimensions of discipline-specific feedback literacy: comparative analysis, expression and communication, mathematical reasoning, monitor and adjust, diagnostic evaluation, and implementation capacity. A sample item is “I can analyze my weaknesses in math learning based on the feedback I receive.” Participants rate items on a 6-point Likert-type scale from 1 (strongly disagree) to 6 (strongly agree). Prior psychometric evaluation supported a hierarchical structure with a second-order general factor and demonstrated strict measurement invariance across grade levels (Chen et al., 2024). In the present study, internal consistency reliability was good (*ω* = 0.85).

#### 2.2.5 Demographic covariates

Demographic covariates were assessed at Time 1 and were entered as observed exogenous predictors in all models. Adolescent age was recorded in years and ranged from 15 to 18. Gender was coded 1 = male and 2 = female. Grade level was coded 1 = Grade 10, 2 = Grade 11, and 3 = Grade 12. The place of origin was coded 1 = urban and 2 = rural. Parental education was coded 1 = high school or below, 2 = associate or bachelor’s degree, and 3 = postgraduate degree. For analysis, categorical covariates were represented with dummy variables using the first category as the reference level, while age was treated as a continuous covariate. Coding was prespecified and applied consistently across all analyses.

### 2.3 Analytic strategies

Analyses followed a prespecified sequence. We first summarized missingness across waves. Consistent with the non-significant Little’s test reported in the Results section, the missing-data pattern was compatible with a missing-completely-at-random mechanism; primary models nonetheless assumed the more general missing-at-random mechanism. All confirmatory factor analyses and structural equation models were estimated in Mplus 8.3 using the robust maximum-likelihood estimator (MLR; ESTIMATOR = MLR), which implements full-information maximum likelihood (FIML) to use all available data under the missing-at-random assumption (Zhang and Savalei, 2020). This robust estimator was specifically selected to ensure valid parameter estimates and standard errors even if the data deviated from the assumption of multivariate normality (Li, 2016). All item indicators were treated as continuous, and categorical covariates were dummy-coded. In the longitudinal mediation models, each construct (T1 school guidance support, T2 major decision-making self-efficacy, T3 feedback literacy behavior, and T1–T3 mathematics discourse feedback skills) was specified as a latent variable measured by its full set of items. Thus, all reported paths and indirect effects are among latent variables rather than based on summed raw scores or CFA-derived factor scores. The scale means reported in Table 1 are simple averages of the corresponding items and are provided solely for descriptive purposes. Descriptive statistics and zero-order correlations were computed in IBM SPSS 25.0 using available-case (pairwise deletion) procedures to align with the reporting in the Results. As a check on univariate normality, skewness coefficients were inspected for each continuous scale score at its respective wave (Ryu, 2011); all absolute skewness values were below 1.0, indicating no substantial departures from normality for the Likert-type variables.

**Table 1:** Descriptive statistics, skewness, and bivariate correlations among study variables.

Variables	*M*	*SD*	Skewness	1	2	3	4	5	6	7	8	9	10	11
1. Gender	–	–	–	1										
2. Age	16.465	0.974	–	−0.022	1									
3. Grade level	–	–	–	−0.015	0.758^**^	1								
4. Place of origin	–	–	–	0.022	−0.034	−0.043^*^	1							
5. Parental education	–	–	–	0.003	−0.015	−0.007	−0.161^**^	1						
6. T1 Student perceptions of school guidance support	2.992	0.947	−0.512	−0.031	0.024	0.024	−0.140^**^	0.161^**^	1					
7. T1 Mathematics discourse feedback skills	3.866	1.185	−0.423	−0.049^*^	0.075^**^	0.094^**^	−0.089^**^	0.089^**^	0.267^**^	1				
8. T2 Major decision-making self-efficacy	3.112	0.847	−0.584	−0.031	0.014	0.013	−0.114^**^	0.110^**^	0.451^**^	0.245^**^	1			
9. T2 Mathematics discourse feedback skills	3.524	1.234	−0.437	−0.039	0.012	0.026	−0.092^**^	0.080^**^	0.281^**^	0.535^**^	0.301^**^	1		
10. T3 Feedback literacy behavior	3.631	1.126	−0.491	−0.043	0.038	0.029	−0.169^**^	0.162^**^	0.350^**^	0.233^**^	0.465^**^	0.285^**^	1	
11. T3 Mathematics discourse feedback skills	3.413	1.309	−0.336	−0.021	0.035	0.022	−0.098^**^	0.101^**^	0.413^**^	0.410^**^	0.472^**^	0.620^**^	0.515^**^	1

*M* and SD are reported for continuous variables; values are omitted for categorical covariates. Coding: gender (1 = male, 2 = female), grade level (1 = Grade 10, 2 = Grade 11, 3 = Grade 12), place of origin (1 = urban, 2 = rural), parental education (1 = high school or below, 2 = associate/bachelor, 3 = postgraduate). Pairwise deletion was used for descriptives and correlations; sample sizes ranged from 1,938 to 2,120 across pairs. ^*^p < 0.05, ^**^p < 0.01.

Preliminary analyses included attrition analyses comparing retained and attrited participants on T1 demographics and study variables, descriptive statistics, bivariate correlations, and diagnostics for multicollinearity among predictors. To evaluate multicollinearity, we inspected correlations among all continuous and dummy-coded predictors that entered the structural models and estimated variance inflation factors (VIFs) and tolerances from auxiliary linear regressions in IBM SPSS 25.0 in which T3 mathematics discourse feedback skills were regressed on T1 school guidance support, T2 major decision-making self-efficacy, T3 feedback literacy behavior, and the demographic covariates (Obrien, 2007). All pairwise correlations among predictors were below |0.80| and all VIF values were ≤2.00 (tolerances ≥ 0.50), indicating that multicollinearity was not a concern. To probe potential common method variance from self-reports, we fitted a common latent factor (CLF) model in Mplus in which a method factor loaded on all indicators, was specified orthogonal to the trait factors, and, where applicable, was constrained to have equal loadings; we compared its fit to the baseline measurement model without the CLF, with both models estimated using the same MLR estimator and FIML missing-data handling described above. This CLF analysis served only as a sensitivity check and was not carried forward to the structural models (Fuller et al., 2016). Measurement modeling preceded structural tests. For mathematics discourse feedback skills, assessed at T1, T2, and T3, we estimated a longitudinal confirmatory factor model with correlated uniqueness among matching indicators across waves and tested measurement invariance in the order of configural, metric, and scalar constraints. Model fit was evaluated using *χ*^2^, Comparative Fit Index (CFI), Tucker-Lewis Index (TLI), Root Mean Square Error of Approximation (RMSEA) with its 90% confidence interval, and Standardized Root Mean Square Residual (SRMR). Decisions about invariance followed commonly used criteria: ΔCFI ≤ 0.010 and ΔRMSEA ≤ 0.015, with ΔSRMR ≤ 0.030 for metric and ≤0.010 for scalar invariance (Abulela et al., 2025).

Structural equation models were then estimated in Mplus 8.3 to test the hypothesized processes. Conceptually, the longitudinal mediation models can be viewed as a traditional three-wave cross-lagged panel model (CLPM) with autoregressive paths for mathematics discourse feedback skills and time-lagged directional paths that follow the hypothesized temporal ordering. We utilized the CLPM framework because it is well-suited for identifying the directionality of effects between contextual supports and skill development while rigorously controlling for the autoregressive stability of each construct over time (Cole and Maxwell, 2003). As in other CLPM applications, the structural coefficients capture time-ordered associations that combine within-student change with stable between-student differences rather than isolating purely within-person processes (Hamaker et al., 2015; Mulder and Hamaker, 2021). First, we tested a time-lagged mediation model corresponding to H1 and H2, in which T1 school guidance support predicted T3 mathematics discourse feedback skills through T2 major decision-making self-efficacy. Mathematics discourse feedback skills at T1 and T2 were included as autoregressive predictors of subsequent skills, and stability paths between adjacent waves were retained. Next, we added T3 feedback literacy behavior as a second mediator to evaluate the additional mediation pathways implied by H3 and H4, including the sequential pathway from T1 school guidance support to T2 major decision-making self-efficacy to T3 feedback literacy behavior to T3 mathematics discourse feedback skills. The direct path from T1 school guidance support to T3 mathematics discourse feedback skills was retained to evaluate partial mediation. Age, gender, grade level, place of origin, and parental education were included as observed covariates and were specified to predict the mediator(s) and the T3 outcome. Within-wave covariances among exogenous variables were freely estimated. In addition to the focal mediational pathways, we also computed the time-ordered indirect effect *via* T2 mathematics discourse feedback skills implied by the autoregressive structure. Indirect and direct effects were evaluated with non-parametric bootstrapping using 5,000 resamples, with bias-corrected 95% confidence intervals obtained *via* the MODEL INDIRECT command. Effects were considered statistically significant when the confidence interval did not include zero. Unless otherwise noted, all tests were two-tailed with *α* = 0.05. We report both unstandardized coefficients (B) and completely standardized estimates (*β*; STDYX), together with model fit indices (Tibbe and Montoya, 2022).

## 3 Results

### 3.1 Attrition analysis

We compared adolescents retained at T3 (Group 1; *n* = 1,938) with those who attrited at T2 and/or T3 (Group 2; *n* = 182) on T1 demographics and study variables. Independent-samples *t*-tests indicated no between-group differences for age, *t*(2118) = −0.37, *p* = 0.711, for student perceptions of school guidance support at T1 across its four dimensions (all *ps* ≥ 0.68), or for mathematics discourse feedback skills at T1 across its six dimensions (all *ps* ≥ 0.30). Levene’s tests supported equality of variances for all outcomes except the monitoring and adjustment dimension at T1; using Welch’s correction for that comparison, the group difference remained non-significant, *t*(221.16) = 0.77, *p* = 0.421.

Chi-square tests for categorical covariates were also non-significant: gender, *χ*^2^(1) = 0.994, *p* = 0.319; grade level, *χ*^2^(2) = 0.473, *p* = 0.789; place of origin, *χ*^2^(1) = 1.847, *p* = 0.174; parental education, *χ*^2^(2) = 1.060, *p* = 0.588. Cramer’s *V* values ranged from 0.015 to 0.030, indicating trivial effects.

Finally, Little’s MCAR test was non-significant, *χ*^2^(33) = 22.74, *p* = 0.910, indicating that the missing-data pattern was consistent with a missing-completely-at-random mechanism. Taken together, these results suggest that attrition is unlikely to bias the study with respect to measured baseline characteristics and are consistent with the assumptions about missing data adopted in the main analyses.

### 3.2 Measurement invariance

We first fit a longitudinal configural model for mathematics discourse feedback skills with the same six indicators specified at T1, T2, and T3 and with correlated uniqueness among matching indicators across waves. The model showed excellent fit, *χ*^2^(114) = 127.32, *p* = 0.186, CFI = 0.999, TLI = 0.999, RMSEA = 0.007, 90% CI [0.000, 0.014], and SRMR = 0.012, supporting an equivalent factor structure over time (see Table 2).

**Table 2:** Longitudinal measurement invariance of mathematics discourse feedback skills (T1–T3).

Model	*χ* ^2^	df	CFI	TLI	RMSEA	SRMR	ΔCFI	ΔRMSEA
Configural	127.32	114	0.999	0.999	0.007	0.012	–	–
Metric	134.97	124	1.000	0.999	0.006	0.014	0.001	−0.001
Scalar	136.69	134	1.000	1.000	0.003	0.014	0.000	−0.003

Decision rules were ΔCFI ≤ 0.010 and ΔRMSEA ≤ 0.015; for SRMR, ≤0.030 when comparing configural to metric and ≤0.010 when comparing metric to scalar. Model specifications included correlated uniqueness among matching indicators across waves; latent means were fixed to zero at T1 and freely estimated at T2 and T3 in the scalar model.

We then constrained corresponding factor loadings to equality across waves to test metric invariance. The metric model also exhibited excellent fit, *χ*^2^(124) = 134.97, *p* = 0.236, CFI = 1.000, TLI = 0.999, RMSEA = 0.006, 90% CI [0.000, 0.013], and SRMR = 0.014. Relative to the configural model, changes in fit were trivial and well within commonly used decision rules (ΔCFI = 0.001 ≤ 0.010; ΔRMSEA = −0.001 ≤ 0.015; ΔSRMR = 0.002 ≤ 0.030), indicating that loadings were invariant across T1–T3.

Finally, we constrained corresponding intercepts to equality across waves while freeing the latent means at T2 and T3 for identification to test scalar invariance. The scalar model exhibited excellent fit, *χ*^2^(134) = 136.69, *p* = 0.419, CFI = 1.000, TLI = 1.000, RMSEA = 0.003, 90% CI [0.000, 0.011], and SRMR = 0.014. Fit changes from the metric model were negligible (ΔCFI = 0.000; ΔRMSEA = −0.003; ΔSRMR = 0.000), meeting recommended thresholds (ΔCFI ≤ 0.010; ΔRMSEA ≤ 0.015; ΔSRMR ≤ 0.010). These results support full scalar invariance, permitting meaningful comparison of latent means over time and the estimation of time-ordered structural relations in subsequent models.

### 3.3 Common method bias

To evaluate whether shared method variance inflated relations among the self-report measures, we used a confirmatory factor approach in Mplus 8.3 (Williams and McGonagle, 2016). We first fit the longitudinal trait-only measurement model across T1, T2, and T3, allowing correlated uniqueness among matching indicators across waves. This model showed excellent fit, *χ*^2^(431) = 469.161, *p* = 0.099, CFI = 0.999, TLI = 0.999, RMSEA = 0.006, 90% CI [0.000, 0.010], SRMR = 0.014. We then estimated an alternative model that added a single CLF that loaded equally on all items and was specified to be orthogonal to the substantive factors; both models were estimated using the robust maximum-likelihood estimator (MLR; ESTIMATOR = MLR) with FIML for missing data, as described in Section 2.3.

Introducing the CLF produced only trivial changes in global fit, *χ*^2^(430) = 460.838, *p* = 0.147, CFI = 0.999, TLI = 0.999, RMSEA = 0.006, 90% CI [0.000, 0.010], and SRMR = 0.014. Although the *χ*^2^ difference was statistically significant, Δ*χ*^2^(1) = 8.323, *p* = 0.004, the incremental fit indices were essentially unchanged (ΔCFI = 0.000; ΔTLI = 0.000; ΔRMSEA = 0.000; ΔSRMR = 0.000). Given the large sample size and the stability of incremental indices, we treat these changes as negligible (Lai and Yoon, 2015). In addition, standardized trait loadings and latent correlations were highly similar across the two models (differences minimal), indicating that the substantive measurement and structural relations were not driven by a general method factor (Geiser and Simmons, 2021). Taken together, these results suggest that common method bias is unlikely to meaningfully compromise the substantive conclusions of the longitudinal models.

### 3.4 Descriptive statistics, normality, and correlations

Table 1 presents descriptive statistics, skewness coefficients, and zero-order correlations for the continuous study variables. Means for all focal constructs clustered near the scale midpoint with adequate dispersion. Skewness values for the scale scores were small in magnitude (|skew| ≤ 0.60), suggesting no serious univariate non-normality for these Likert-type variables. Accordingly, the assumption of approximate normality required for the MLR estimator was judged to be adequately met. Moreover, the use of a robust estimator (MLR) together with bias-corrected bootstrapping for indirect effects further mitigates any potential influence of minor deviations from normality on the inferential results. Consistent with the multicollinearity diagnostics, the largest bivariate correlation among predictors was 0.76 (between age and grade level), which is below common cutoffs for problematic multicollinearity, and auxiliary regression models yielded VIF values no greater than 2.00 (tolerances ≥ 0.50), further indicating that multicollinearity was not a concern in the structural models. As expected, T1 school guidance support correlated positively with T2 major decision-making self-efficacy (*r* = 0.45, *p* < 0.01) and with T3 feedback literacy behavior (*r* = 0.35, *p* < 0.01), as well as with mathematics discourse feedback skills across waves (*rs* ranged from 0.27 to 0.41, all *ps* < 0.01). T2 major decision-making self-efficacy was positively related to T3 feedback literacy behavior (*r* = 0.47, *p* < 0.01) and to T3 mathematics discourse feedback skills (*r* = 0.47, *p* < 0.01). At T3, feedback literacy behavior correlated moderately with mathematics discourse feedback skills (*r* = 0.52, *p* < 0.01). Mathematics discourse feedback skills showed small-to-moderate stability across time (adjacent-wave *rs* = 0.54 and 0.62; from T1 to T3, *r* = 0.41; all *ps* < 0.01).

### 3.5 Mediating effects of major decision-making self-efficacy

**Figure 2:**
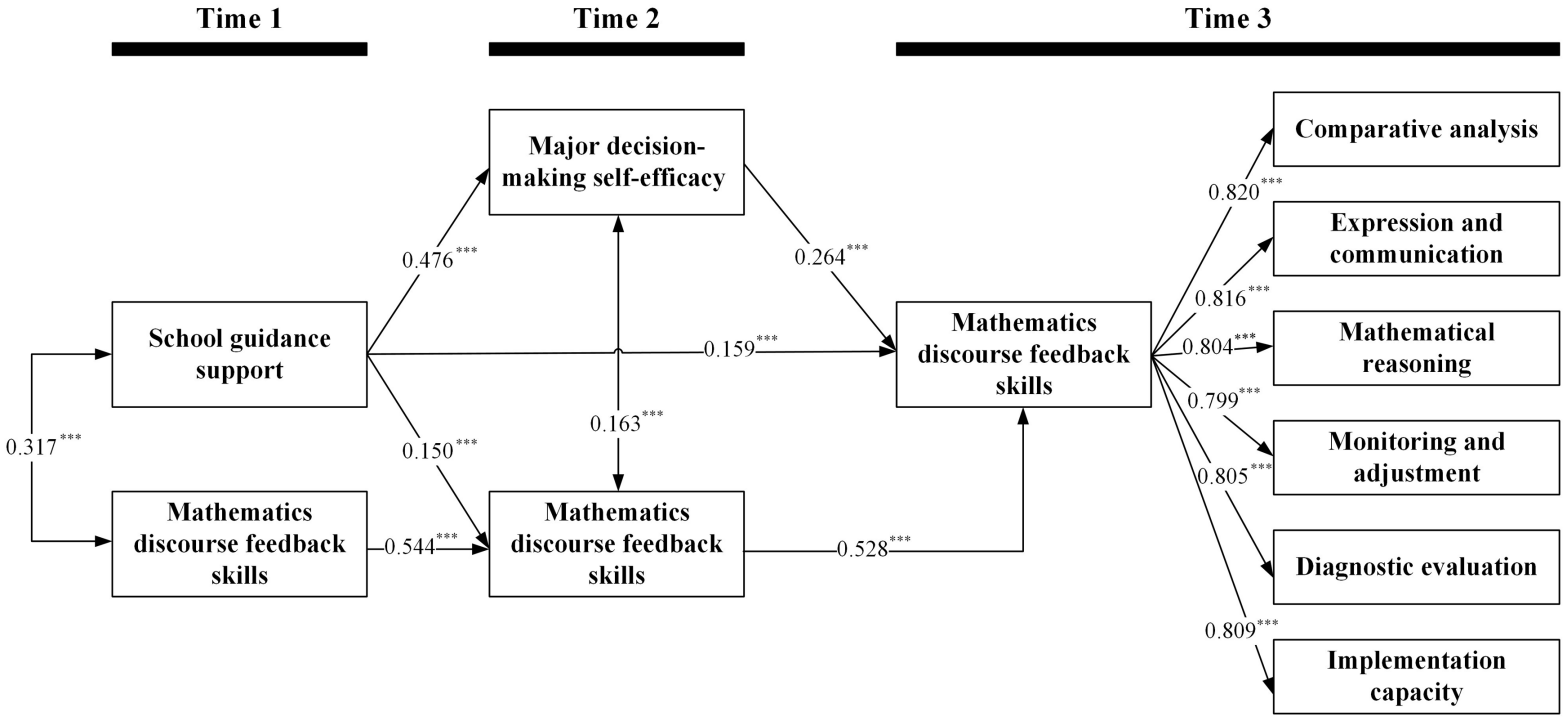
Single-mediator longitudinal structural equation model linking T1 school guidance support to T3 mathematics discourse feedback skills *via* T2 major decision-making self-efficacy. ***p < 0.001, this indicates the level of statistical significance for the path coefficients shown in the models.

The single-mediator longitudinal SEM (Table 3 and Figure 2) showed excellent fit, *χ*^2^(435) = 534.26, CFI = 0.997, TLI = 0.996, RMSEA = 0.010 (90% CI [0.007, 0.013]), and SRMR = 0.031. Consistent with the mediation hypothesis, T1 school guidance support positively predicted T2 major decision-making self-efficacy (*β* = 0.476, 95% CI [0.434, 0.512], *p* < 0.001), and T2 major decision-making self-efficacy positively predicted T3 mathematics discourse feedback skills (*β* = 0.264, 95% CI [0.235, 0.293], *p* < 0.001). With prior mathematics discourse feedback skills and demographic covariates controlled, the indirect effect from T1 school guidance support to T3 mathematics discourse feedback skills through T2 major decision-making self-efficacy was significant (*β* = 0.126, SE = 0.009, 95% CI [0.107, 0.142], *p* < 0.001). These findings confirm H2, which specified that T2 major decision-making self-efficacy mediates the association between T1 school guidance support and T3 mathematics discourse feedback skills.

**Table 3:** Indirect, direct, and total effects from T1 school guidance support to T3 mathematics discourse feedback skills.

Effect	Bias-corrected bootstrapped estimates for the effects		
	*B* (SE)	*β* (SE)	95% CI (*β*)
Direct pathway			
T1 Student perceptions of school guidance support → T3 Mathematics discourse feedback skills	0.222 (0.030)	0.159^***^ (0.021)	[0.120, 0.200]
Indirect pathways			
T1 Student perceptions of school guidance support → T2 Major decision-making self-efficacy → T3 Mathematics discourse feedback skills	0.176 (0.013)	0.126^***^ (0.009)	[0.107, 0.142]
T1 Student perceptions of school guidance support →T2 Mathematics discourse feedback skills → T3 Mathematics discourse feedback skills	0.110 (0.018)	0.079^***^ (0.013)	[0.054, 0.104]

Estimates are from the single-mediator longitudinal SEM with 5,000 bias-corrected bootstrap resamples. Completely standardized coefficients (*β*) are reported alongside unstandardized coefficients (*B*). Models controlled for baseline (T1) and intermediate (T2) mathematics discourse feedback skills and demographic covariates; within-wave covariances among exogenous variables were freely estimated. ^***^p < 0.001.

The direct effect from T1 school guidance support to T3 mathematics discourse feedback skills remained positive and significant (*β* = 0.159, SE = 0.021, 95% CI [0.120, 0.200], *p* < 0.001), indicating partial mediation and supporting H1 regarding a positive influence of T1 school guidance support on later mathematics discourse feedback skills. For completeness, a secondary time-ordered indirect pathway through T2 mathematics discourse feedback skills was also significant (*β* = 0.079, SE = 0.013, 95% CI [0.054, 0.104], *p* < 0.001), consistent with strong stability from T2 to T3 (*β* = 0.528, *p* < 0.001). The total standardized effect from T1 school guidance support to T3 mathematics discourse feedback skills was *β* = 0.363 (95% CI [0.323, 0.410]), and *β* = 0.205 (95% CI [0.174, 0.233]) was transmitted through all indirect paths combined. The focal indirect pathway *via* major decision-making self-efficacy accounted for approximately 34.8% of the total standardized effect (0.126/0.363), whereas all indirect paths together accounted for about 56.5% (0.205/0.363).

### 3.6 Sequential mediation via T2 major decision-making self-efficacy and T3 feedback literacy behavior

**Figure 3:**
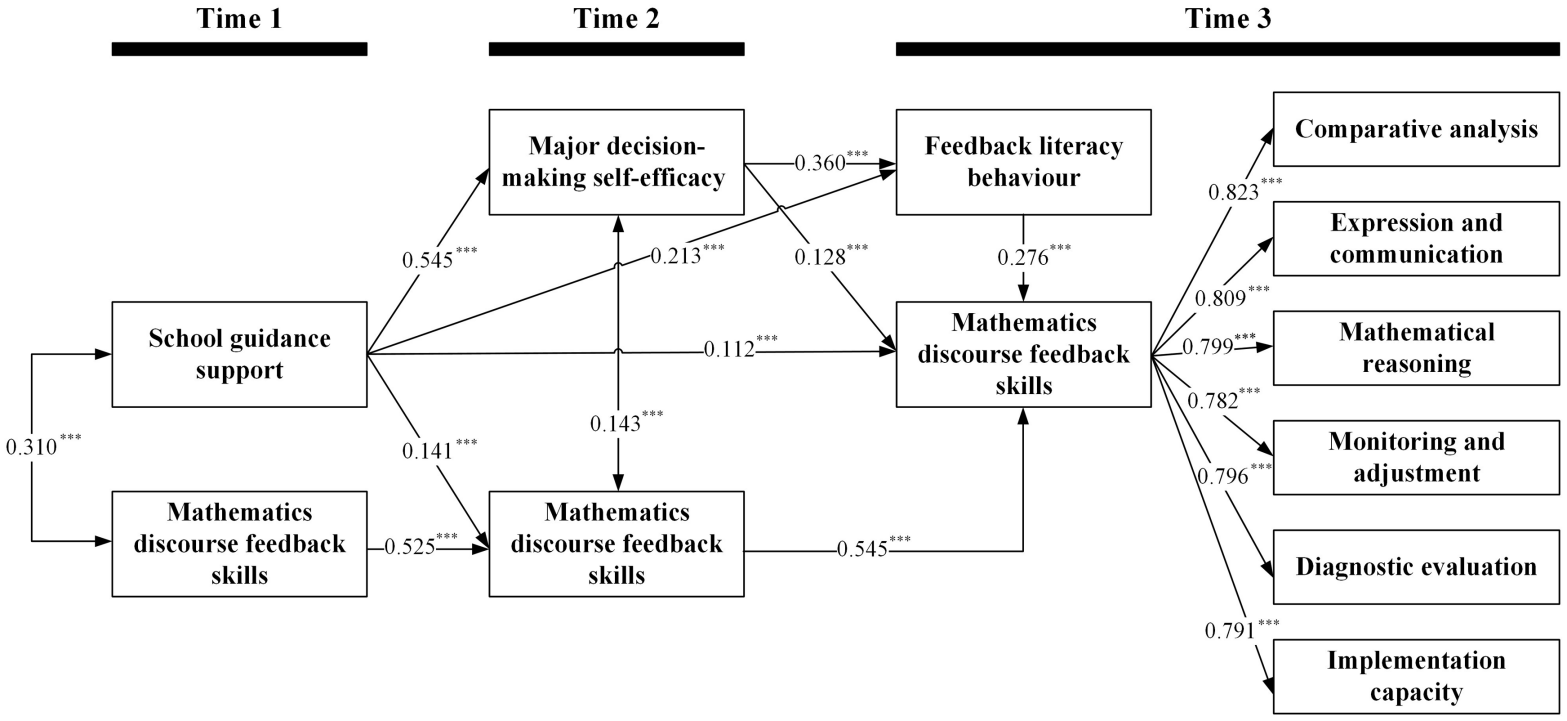
Three-wave sequential-mediation structural equation model linking T1 school guidance support to T3 mathematics discourse feedback skills *via* T2 major decision-making self-efficacy and T3 feedback literacy behavior. ***p < 0.001, this indicates the level of statistical significance for the path coefficients shown in the models.

We estimated a three-wave sequential mediation model (Tables 4, 5 and Figure 3) in which T1 school guidance support predicted T3 mathematics discourse feedback skills through T2 major decision-making self-efficacy and T3 feedback literacy behavior, while controlling for prior mathematics discourse feedback skills at T1 and T2 and all prespecified covariates. Model fit was excellent, *χ*^2^(603) = 779.79, CFI = 0.990, TLI = 0.989, RMSEA = 0.017 (90% CI [0.013, 0.020]), and SRMR = 0.047; models were estimated using MLR with FIML for missing data, as described in Section 2.3.

**Table 4:** Sequential mediation of the association between T1 school guidance support and T3 mathematics discourse feedback skills.

Predictors	T2 Major decision-making self-efficacy (*R*^2^ = 0.302)		T3 Feedback literacy behavior (*R*^2^ = 0.293)		T2 Mathematics discourse feedback skills (*R*^2^ = 0.343)		T3 Mathematics discourse feedback skills (*R*^2^ = 0.597)	
	*B*	*β* (SE)	*B*	*β* (SE)	*B*	*β* (SE)	*B*	*β* (SE)
T1 School guidance support	0.539	0.545^***^ (0.029)	0.277	0.213^***^ (0.043)	0.184	0.141^***^ (0.033)	0.154	0.112^***^ (0.033)
T2 Major decision-making self-efficacy			0.473	0.360^***^ (0.040)			0.177	0.128^***^ (0.035)
T3 Feedback literacy behavior							0.291	0.276^***^ (0.033)
T1 Mathematics discourse feedback skills					0.543	0.525^***^ (0.028)		
T2 Mathematics discourse feedback skills							0.576	0.545^***^ (0.026)

Column headers give *R*^2^ for each endogenous latent. ^***^p < 0.001. Blank cells indicate paths not specified.

**Table 5:** Indirect effects from T1 school guidance support to T3 mathematics discourse feedback skills in the sequential mediation model.

Indirect effects	Bias-corrected bootstrapped estimates for the effects			
	*B*	*β*	SE	95% CI
T1 School guidance support → T2 Major decision-making self-efficacy → T3 Mathematics discourse feedback skills	0.096	0.070	0.020	[0.033, 0.109]
T1 School guidance support → T2 Mathematics discourse feedback skills → T3 Mathematics discourse feedback skills	0.106	0.077	0.018	[0.041, 0.112]
T1 School guidance support → T3 Feedback literacy behavior → T3 Mathematics discourse feedback skills	0.081	0.059	0.014	[0.034, 0.088]
T1 School guidance support → T2 Major decision-making self-efficacy → T3 Feedback literacy behavior → T3 Mathematics discourse feedback skills	0.074	0.054	0.010	[0.037, 0.076]

*B* = unstandardized effect; *β* = completely standardized effect (STDYX); SE = bootstrap standard error; 95% CI = bias-corrected bootstrap confidence interval.

The total effect of T1 school guidance support on T3 mathematics discourse feedback skills was positive and statistically significant (*β* = 0.371, SE = 0.031, 95% CI [0.308, 0.430], *p* < 0.001). The direct effect remained significant after mediators were included (*β* = 0.112, SE = 0.033, 95% CI [0.046, 0.177], *p* = 0.001), supporting H1. As hypothesized, the indirect effect *via* T2 major decision-making self-efficacy was significant (*β* = 0.070, SE = 0.020, 95% CI [0.033, 0.109], *p* < 0.001), confirming H2; the indirect effect *via* T3 feedback literacy behavior was also significant (*β* = 0.059, SE = 0.014, 95% CI [0.034, 0.088], *p* < 0.001), supporting H3. Critically, the sequential indirect effect from T1 school guidance support → T2 major decision-making self-efficacy → T3 feedback literacy behavior → T3 mathematics discourse feedback skills was significant (*β* = 0.054, SE = 0.010, 95% CI [0.037, 0.076], *p* < 0.001), providing affirmative evidence for H4. In addition, a time-ordered indirect effect *via* T2 mathematics discourse feedback skills (controlling for T1 skills) was significant (*β* = 0.077, SE = 0.018, 95% CI [0.041, 0.112], *p* < 0.001), indicating that improvements in skills at T2 also transmitted the influence of guidance support to T3 skills. For completeness, T2 major decision-making self-efficacy predicted T3 mathematics discourse feedback skills both directly (*β* = 0.128, SE = 0.035, *p* < 0.001) and indirectly *via* T3 feedback literacy behavior (*β* = 0.099, SE = 0.017, 95% CI [0.071, 0.136], *p* < 0.001).

## 4 Discussion

### 4.1 Summary of findings

This three-wave study examined whether T1 school guidance support predicts T3 mathematics discourse feedback skills and whether this association is accounted for by T2 major decision-making self-efficacy and T3 feedback literacy behavior, while adjusting for prior levels of the outcome and demographic covariates. Consistent with our expectations, school guidance support at T1 was positively related to mathematics discourse feedback skills at T3 after controls, supporting H1. The association was partly transmitted through each mediator considered separately: students reporting stronger guidance support displayed higher decision-making self-efficacy at T2, which was related to stronger skills at T3 (H2), and they reported more feedback literacy behavior at T3, which was also associated with stronger skills (H3). Importantly, the hypothesized sequential pathway received support (H4): T1 guidance support fostered T2 decision-making self-efficacy, which was linked to greater T3 feedback literacy behavior and, in turn, to higher T3 mathematics discourse feedback skills. The pattern of results held with robust estimation and bias-corrected bootstrapping and was obtained while accounting for autoregressive stability from T1 to T2 and from T2 to T3. Together, these findings delineate a coherent mechanism through which school-level guidance practices promote the development of discipline-specific feedback skills *via* motivational (self-efficacy) and behavioral (feedback literacy) processes.

### 4.2 Theoretical implications

The present findings clarify how school-level supports translate into discipline-specific learning capacities through motivational and behavioral mechanisms. By demonstrating that T1 school guidance support relates to T3 mathematics discourse feedback skills both directly and indirectly *via* T2 major decision-making self-efficacy and T3 feedback literacy behavior, the study identifies a coherent temporal chain from contextual affordances to agentic beliefs, to feedback-related enactment, and finally to domain-specific skill.

Notably, the direct path from T1 school guidance support to T3 mathematics discourse feedback skills remained significant after accounting for T2 major decision-making self-efficacy, T3 feedback literacy behavior, and intermediate mathematics discourse feedback skills. This residual association likely reflects additional mechanisms and contextual influences that were not explicitly modeled in the current study. For example, guidance support in these schools is closely tied to course placement, access to advanced mathematics tracks, and opportunities to participate in examination-focused enrichment activities, all of which may shape students’ exposure to high-quality mathematical discourse independently of their self-efficacy and feedback literacy behaviors (Donaldson et al., 2017). Guidance staff and homeroom teachers may also influence classroom norms, teacher expectations, and the structuring of practice opportunities in ways that promote discourse-based feedback use beyond the specific mediators that we assessed (Raynham and Jinks, 2022). Furthermore, unmeasured motivational and socio-cognitive constructs, such as achievement goal orientations, perceived belonging in mathematics, or perceived expectations from significant others, may operate in parallel with the tested mediators (Lin et al., 2025). The remaining direct effect should therefore be interpreted as a composite of these unobserved pathways rather than as evidence of an unmediated or purely structural impact of guidance support on mathematics discourse feedback skills.

First, the results elaborate on social cognitive theory and social cognitive career theory by specifying school guidance support as a proximal contextual source that shapes efficacy beliefs relevant to academic decision-making (Riley et al., 2015; Wang et al., 2022). In this account, structured informational resources, credible modeling, and persuasive encouragement are not merely ancillary inputs; they are the conditions under which students come to appraise themselves as capable of navigating consequential choices (McGeough and Rudick, 2018). The observed mediation indicates that the influence of guidance support on later academic performance capacities is carried, in part, by strengthened self-efficacy that orients students toward approach goals, persistence under uncertainty, and strategic engagement (Zhong et al., 2023). Second, the study advances the feedback literacy framework by locating feedback literacy behavior in a longitudinal sequence that culminates in mathematics discourse feedback skills. Feedback literacy behavior functions as the behavioral conduit that converts evaluative information into action. Positioning this conduit after efficacy in time suggests a motivational–behavioral alignment: students who feel efficacious are more willing to seek, interpret, and use feedback, and those behaviors, repeated across feedback-rich episodes, consolidate into higher-level discourse skills that are specific to mathematics (Zhang, 2025). Third, the findings integrate self-regulated learning perspectives with dialogic views of classroom discourse (Callan and Cleary, 2019). Guidance practices that clarify goals and standards, provide exemplars, and normalize conferences with teachers and peers create conditions that support forethought, monitoring during performance, and reflective revision (Chung et al., 2021). The sequential pattern indicates that these conditions operate through students’ beliefs and behaviors rather than bypassing them, reinforcing theoretical accounts that place regulation at the core of durable skill growth within disciplinary discourse (Russell et al., 2022). Fourth, the distinction between feedback literacy behavior and mathematics discourse feedback skills has conceptual value. The former reflects general repertoires for engaging with evaluative information across contexts, whereas the latter indexes what students can enact in a specific disciplinary conversation (Nieminen and Carless, 2023). Showing that guidance support propagates through both levels suggests a nested architecture in which domain-general behavioral repertoires scaffold domain-specific competencies. This distinction encourages future models to represent multiple layers of skill expression rather than treating feedback-related capacities as monolithic (Ren et al., 2025). Beyond individual processes, the findings need to be situated within the institutional and cultural features of Chinese upper-secondary education. Senior high schools in mainland China operate within an examination-driven system in which the gaokao and related entrance examinations strongly structure students’ academic trajectories, and major and institutional choices are often framed as high-stakes, relatively irreversible decisions (Zivin et al., 2020). Within this context, school guidance support typically combines counselor-led information sessions, class-based guidance lessons, and teacher mentoring that are tightly coupled to subject-stream selection, major choice, and preparation for competitive examinations (Xiong et al., 2023). From a social cognitive career theory perspective, such configurations make guidance services a salient contextual affordance that can function both as support and as a gatekeeping mechanism, shaping efficacy beliefs, outcome expectations, and perceived barriers in particularly potent ways (Lent and Brown, 2019). In more decentralized or less exam-driven systems, guidance relationships may be less directive and more exploratory, and the balance of influence between school-level supports, family resources, and labor-market cues may differ (Magee et al., 2022). Comparative research is therefore needed to examine whether the same pattern of supports → efficacy → feedback-related behaviors → disciplinary skills holds in other policy regimes, and whether the strength of these pathways varies across guidance systems with different levels of selectivity and stakes. Finally, the time-ordered evidence highlights the importance of sequencing supports. The pattern implies that interventions may be most effective when schools first cultivate decision-making self-efficacy, then engineer repeated opportunities for feedback literacy behavior, and only then expect measurable growth in discipline-specific discourse skills. Theory should, therefore, consider temporal design features as core elements rather than implementation details.

### 4.3 Practical implications for guidance and instruction

The findings suggest a practical sequence: build T2 decision-making self-efficacy through guidance, cultivate T3 feedback-using behaviors in instruction, and then expect growth in mathematics discourse feedback skills. Schools should time guidance activities early in the cycle, use brief progress checks on self-efficacy and feedback literacy mid-cycle, and assess discourse-based revision and reasoning later (Smit et al., 2023a). This alignment keeps proximal targets visible and actionable rather than treating discipline-specific skills as the only outcome.

For guidance and classroom practice, combine standard routines with a few less common, high-leverage tactics. In guidance, run short decision studios that rehearse option generation, evidence appraisal, and justification; use pre-mortem planning where students identify how a major choice could fail and design safeguards; rotate near-peer advisory panels that model authentic choice processes; award a micro-credential when students complete a documented decision plan with criteria and contingencies (Ahsan et al., 2023; Krishna et al., 2024). In mathematics instruction, issue a feedback passport that requires students to log specific requests and actions each week; hold live error clinics in which students bring a mistaken solution and narrate the repair; use blind peer critique with justification tokens so comments must reference criteria; require audio or screen-capture revision memos that demonstrate how feedback changed a solution; adopt two-stage problems that release scores only after a short action plan is submitted; pilot mini oral checkpoints where learners explain one reasoning step and request a targeted clarification (Patchan et al., 2022). Coordinate these efforts with shared tools such as concise rubrics and exemplar libraries, provide protected time for revision to support students with lower initial self-efficacy, and track change with brief observational rubrics for discourse participation alongside self-reports (Gotch et al., 2021).

### 4.4 Limitations and directions for future research

Several limitations qualify the interpretations. All focal constructs were assessed *via* student self-reports collected within the same sessions, which introduces the possibility of shared method variance and common rater effects, even though the CLF sensitivity analysis suggested that a general method factor did not substantively distort the measurement structure or structural relations (Fuller et al., 2016; Podsakoff et al., 2003). The three-wave design with fixed intervals constrains inferences about short-cycle change, and the observational models cannot establish causality despite longitudinal ordering, statistical controls, and robustness checks. Moreover, the structural models correspond to a traditional latent-variable CLPM. By design, this framework does not decompose within-student fluctuations from stable between-student differences, so the time-lagged coefficients should be interpreted as directional, time-ordered associations rather than definitive within-person causal effects. Recent methodological work shows that random-intercept (RI) CLPMs and multilevel extensions can separate within-student change from stable traits and account for clustering (Hamaker et al., 2015; Mulder and Hamaker, 2021). Our design included only one measurement occasion for each mediator, which precluded specifying a full RI-CLPM for all constructs (Falkenström et al., 2022). Mathematics discourse feedback skills were not triangulated with performance tasks, observational ratings, or student work samples, so the outcome reflects perceived rather than directly observed discourse feedback skills in mathematics classrooms (Han et al., 2020). At present, empirical evidence directly linking MDFSS scores to objective indices of mathematics achievement, such as test scores, course grades, or rubric-scored performance on curriculum-aligned tasks, is still emerging (Bishop, 2021; Chen et al., 2020). Accordingly, the present findings should be interpreted as speaking primarily to growth in students’ perceived discourse feedback skills rather than to demonstrated gains in mathematics performance. The sample comprised Chinese senior high schools operating in a highly examination-driven system, which may limit direct generalization to more decentralized or less competitive educational contexts, and unmeasured classroom or school factors may also have contributed to the associations we observed (Hu and Qian, 2025).

Future studies should strengthen causal inference through multi-site experiments or quasi-experiments that manipulate guidance, support, and feedback routines (Rakoczy et al., 2019). Multilevel or random-intercept cross-lagged models can then be used to examine within-student processes while accounting for clustering, and intensive longitudinal designs such as experience sampling or learning analytics can capture day-to-day feedback use (Li and Wang, 2022). In addition to self-report scales, multi-method and multi-informant assessment batteries that include rubric-scored student work in mathematics, transcripts or recordings of classroom discourse, systematic classroom observations of feedback episodes, teacher ratings of students’ feedback-related behaviors, and artifact analysis would allow stronger tests of whether the longitudinal relations observed here replicate when mathematics discourse feedback skills are indexed by performance-based evidence rather than perceptions alone and would further validate and extend the outcome measures (Joyce et al., 2018). Researchers should test alternative time lags, reciprocal pathways, and additional mediators and moderators, and conduct cross-cultural replications. Person-centered analyses can identify subgroups that follow distinct developmental pathways, and mechanism-focused trials can estimate the optimal sequencing and dosage of supports (Giesbrecht et al., 2022).

### 4.5 Conclusion

This study traced how school guidance support at T1 relates to mathematics discourse feedback skills at T3 through a motivational and behavioral route. The results delineate a time-ordered mechanism where guidance acts not merely as a distal resource but as a catalyst that sequentially strengthens agentic beliefs and enables feedback-using practices. It is important to emphasize, however, that these conclusions are drawn from observational data and reflect students’ self-reported confidence in their regulatory enactments (perceived competence) rather than objectively verified performance gains.

Nevertheless, the robust statistical associations provide specific evidence-based directives for educational practice. First, the significant pathway from guidance support to major decision-making self-efficacy (*β* = 0.476) suggests that schools should prioritize early guidance interventions to build students’ confidence before they enter high-stakes assessment periods. Second, the finding that feedback literacy behavior significantly predicts subsequent mathematics discourse feedback skills (*β* = 0.276), even after controlling for autoregressive stability, indicates that instructional designs must go beyond merely providing comments; they must explicitly engineer opportunities for students to seek, interpret, and enact feedback. Theoretically, these data integrate social cognitive accounts with feedback literacy perspectives, confirming that contextual supports translate into domain-specific discipline skills primarily by fostering the “will” (efficacy) and the “skill” (literacy behavior) to engage with evaluative information.

## Data Availability

The raw data supporting the conclusions of this article will be made available by the authors without undue reservation.

## References

[ref1] Abulela M. A. A. Nickodem K. Rodriguez M. C. (2025). Measurement invariance and cohort trends for social and emotional learning measures across four statewide administrations: conventional fit statistics versus the RMSEAD. J. Psychoeduc. Assess. 43, 178–198. doi: 10.1177/07342829241302238

[ref2] Ahsan K. Akbar S. Kam B. Abdulrahman M. D.-A. (2023). Implementation of micro-credentials in higher education: a systematic literature review. Educ. Inf. Technol. 28, 13505–13540. doi: 10.1007/s10639-023-11739-z

[ref3] Bandura A. (2023). “Cultivate self-efficacy for personal and organizational effectiveness” in Principles of organizational behavior (Hoboken, NJ: John Wiley & Sons, Ltd), 113–135.

[ref4] Bishop J. P. (2021). Responsiveness and intellectual work: features of mathematics classroom discourse related to student achievement. J. Learn. Sci. 30, 466–508. doi: 10.1080/10508406.2021.1922413

[ref5] Biza I. Nardi E. Zachariades T. (2018). “Competences of mathematics teachers in diagnosing teaching situations and offering feedback to students: specificity, consistency and reification of pedagogical and mathematical discourses” in Diagnostic competence of mathematics teachers: Unpacking a complex construct in teacher education and teacher practice. eds. Leuders T. Philipp K. Leuders J. (Cham: Springer International Publishing), 55–78.

[ref6] Bouwer R. Dirkx K. (2023). The eye-mind of processing written feedback: unraveling how students read and use feedback for revision. Learn. Instr. 85:101745. doi: 10.1016/j.learninstruc.2023.101745

[ref7] Callan G. L. Cleary T. J. (2019). Examining cyclical phase relations and predictive influences of self-regulated learning processes on mathematics task performance. Metacogn. Learn. 14, 43–63. doi: 10.1007/s11409-019-09191-x

[ref8] Callan G. L. Rubenstein L. D. Ridgley L. M. Neumeister K. S. Finch M. E. H. (2021). Self-regulated learning as a cyclical process and predictor of creative problem-solving. Educ. Psychol. 41, 1139–1159. doi: 10.1080/01443410.2021.1913575

[ref9] Capa-Aydin Y. Uzuntiryaki-Kondakci E. Ceylandag R. (2018). The relationship between vicarious experience, social persuasion, physiological state, and chemistry self-efficacy: the role of mastery experience as a mediator. Psychol. Sch. 55, 1224–1238. doi: 10.1002/pits.22201

[ref10] Carless D. Boud D. (2018). The development of student feedback literacy: enabling uptake of feedback. Assess. Eval. High. Educ. 43, 1315–1325. doi: 10.1080/02602938.2018.1463354

[ref11] Carless D. Winstone N. (2023). Teacher feedback literacy and its interplay with student feedback literacy. Teach. High. Educ. 28, 150–163. doi: 10.1080/13562517.2020.1782372

[ref12] Carless D. Young S. (2024). Feedback seeking and student reflective feedback literacy: a sociocultural discourse analysis. High. Educ. 88, 857–873. doi: 10.1007/s10734-023-01146-1

[ref13] Chen G. Chan C. K. K. Chan K. K. H. Clarke S. N. Resnick L. B. (2020). Efficacy of video-based teacher professional development for increasing classroom discourse and student learning. J. Learn. Sci. 29, 642–680. doi: 10.1080/10508406.2020.1783269, 41307611

[ref14] Chen H. Tang S. Zhang S. Xu J. Wang G. (2024). Development and validation of the high school students’ mathematics discourse feedback skills scale (MDFSS). Curr. Psychol. 43, 30290–30305. doi: 10.1007/s12144-024-06578-1

[ref15] Chinpakdee M. (2025). Talking feedback: fostering student feedback literacy through a dialogic approach. RELC J.:00336882251346206. doi: 10.1177/00336882251346206

[ref16] Chung H. Q. Chen V. Olson C. B. (2021). The impact of self-assessment, planning and goal setting, and reflection before and after revision on student self-efficacy and writing performance. Read. Writ. 34, 1885–1913. doi: 10.1007/s11145-021-10186-x

[ref17] Cole D. A. Maxwell S. E. (2003). Testing mediational models with longitudinal data: questions and tips in the use of structural equation modeling. J. Abnorm. Psychol. 112, 558–577. doi: 10.1037/0021-843X.112.4.558, 14674869

[ref18] Corwin Z. B. Venegas K. M. Oliverez P. M. Colyar J. E. (2004). School counsel: how appropriate guidance affects educational equity. Urban Educ. 39, 442–457. doi: 10.1177/0042085904265107

[ref19] Curtis K. Chong S. W. Kong M. S. (2025). A qualitative synthesis of research into the use of exemplars in the English for academic purposes context to develop student feedback literacy. Res. Synth. Appl. Linguist. 1, –339. doi: 10.1080/29984475.2025.2495271

[ref20] Dawson P. Yan Z. Lipnevich A. Tai J. Boud D. Mahoney P. (2024). Measuring what learners do in feedback: the feedback literacy behaviour scale. Assess. Eval. High. Educ. 49, 348–362. doi: 10.1080/02602938.2023.2240983

[ref21] de Kleijn R. A. M. (2023). Supporting student and teacher feedback literacy: an instructional model for student feedback processes. Assess. Eval. High. Educ. 48, 186–200. doi: 10.1080/02602938.2021.1967283

[ref22] de Lange T. Wittek A. L. (2022). Analysing the constitution of trust in peer-based teacher mentoring groups – a sociocultural perspective. Teach. High. Educ. 27, 337–351. doi: 10.1080/13562517.2020.1724936

[ref23] Demanet J. Van Houtte M. (2024). “Significant others as environmental resources: toward a sociological refinement of social cognitive career theory” in Young people’s career development and wellbeing: an enquiry across national education systems based on longitudinal data. eds. Knight E. Okay-Somerville B. (Cham: Springer International Publishing), 135–158.

[ref24] Donaldson M. L. LeChasseur K. Mayer A. (2017). Tracking instructional quality across secondary mathematics and English language arts classes. J. Educ. Change 18, 183–207. doi: 10.1007/s10833-015-9269-x

[ref25] Erath K. Prediger S. Quasthoff U. Heller V. (2018). Discourse competence as important part of academic language proficiency in mathematics classrooms: the case of explaining to learn and learning to explain. Educ. Stud. Math. 99, 161–179. doi: 10.1007/s10649-018-9830-7

[ref26] Falkenström F. Solomonov N. Rubel J. A. (2022). How to model and interpret cross-lagged effects in psychotherapy mechanisms of change research: a comparison of multilevel and structural equation models. J. Consult. Clin. Psychol. 90, 446–458. doi: 10.1037/ccp0000727, 35604748 PMC9245087

[ref27] Fort I. Cerqueira E. (2025). Impact of social support dimensions on academic self-efficacy, outcome expectations, and persistence intentions of students from diverse disciplines. Int. J. Educ. Vocat. Guid. doi: 10.1007/s10775-025-09746-2

[ref28] Fuller C. M. Simmering M. J. Atinc G. Atinc Y. Babin B. J. (2016). Common methods variance detection in business research. J. Bus. Res. 69, 3192–3198. doi: 10.1016/j.jbusres.2015.12.008

[ref29] Gao X. Brown G. T. L. (2023). The relation of students’ conceptions of feedback to motivational beliefs and achievement goals: comparing Chinese international students to New Zealand domestic students in higher education. Educ. Sci. 13, 1090. doi: 10.3390/educsci13111090

[ref30] Geiser C. Simmons T. G. (2021). Do method effects generalize across traits (and what if they don’t)? J. Pers. 89, 382–401. doi: 10.1111/jopy.12625, 33586182

[ref31] Giesbrecht G. F. Letourneau N. Dewey D. (2022). Latent class trajectories of infant temperament and associations with problem behavior at two years of age. Dev. Psychopathol. 34, 69–84. doi: 10.1017/S0954579420000991, 32938514

[ref32] Gotch C. M. Poppen M. I. Razo J. E. Modderman S. (2021). Examination of teacher formative assessment self-efficacy development across a professional learning experience. Teach. Dev. 25, 534–548. doi: 10.1080/13664530.2021.1943503

[ref33] Guo J. Abu Talib M. Guo B. Ren J. Liu J. (2025). The mediating role of satisfaction with life and social interaction anxiety in the relationship between loneliness and regulatory emotional self-efficacy. Behav. Sci. 15, 392. doi: 10.3390/bs15030392, 40150286 PMC11939567

[ref34] Hamaker E. L. Kuiper R. M. Grasman R. P. (2015). A critique of the cross-lagged panel model. Psychol. Methods 20, 102–116. doi: 10.1037/a0038889, 25822208

[ref35] Han F. Pardo A. Ellis R. A. (2020). Students’ self-report and observed learning orientations in blended university course design: how are they related to each other and to academic performance? J. Comput. Assist. Learn. 36, 969–980. doi: 10.1111/jcal.12453

[ref36] Heron M. Medland E. Winstone N. Pitt E. (2023). Developing the relational in teacher feedback literacy: exploring feedback talk. Assess. Eval. High. Educ. 48, 172–185. doi: 10.1080/02602938.2021.1932735

[ref37] Hu J. Qian S. (2025). Correlations and comparisons of teacher expectations achievement motivation academic achievement and creativity. Front. Psychol. 16:1516405. doi: 10.3389/fpsyg.2025.1516405, 40918279 PMC12408676

[ref38] Jin F. Maheshi B. Martinez-Maldonado R. Gašević D. Tsai Y.-S. (2024). Scaffolding feedback literacy: designing a feedback analytics tool with students. J. Learn. Anal. 11, 123–137. doi: 10.18608/jla.2024.8339

[ref39] Joughin G. Boud D. Dawson P. Tai J. (2021). What can higher education learn from feedback seeking behaviour in organisations? Implications for feedback literacy. Assess. Eval. High. Educ. 46, 80–91. doi: 10.1080/02602938.2020.1733491

[ref40] Joyce J. Gitomer D. H. Iaconangelo C. J. (2018). Classroom assignments as measures of teaching quality. Learn. Instr. 54, 48–61. doi: 10.1016/j.learninstruc.2017.08.001, 29615831 PMC5862095

[ref41] Keiler L. S. Diotti R. Hudon K. Ransom J. C. (2020). The role of feedback in teacher mentoring: how coaches, peers, and students affect teacher change. Mentor. Tutor. Partnersh. Learn. 28, 126–155. doi: 10.1080/13611267.2020.1749345

[ref42] Ketonen L. Nieminen P. Hähkiöniemi M. (2020). The development of secondary students’ feedback literacy: peer assessment as an intervention. J. Educ. Res. 113, 407–417. doi: 10.1080/00220671.2020.1835794

[ref43] Khiat H. Vogel S. (2022). A self-regulated learning management system: enhancing performance, motivation and reflection in learning. J. Univ. Teach. Learn. Pract. 19, 42–58. doi: 10.53761/1.19.2.4

[ref44] Koichu B. (2019). “A discursively oriented conceptualization of mathematical problem solving” in Problem solving in mathematics instruction and teacher professional development. eds. Felmer P. Liljedahl P. Koichu B. (Cham: Springer International Publishing), 43–66.

[ref45] Krishna L. K. R. Hamid N. A. B. A. Phua G. L. G. Mason S. Hill R. Lim C. . (2024). Peer mentorship and professional identity formation: an ecological systems perspective. BMC Med. Educ. 24:1007. doi: 10.1186/s12909-024-05992-0, 39278932 PMC11403841

[ref46] Labuhn A. S. Zimmerman B. J. Hasselhorn M. (2010). Enhancing students’ self-regulation and mathematics performance: the influence of feedback and self-evaluative standards. Metacogn. Learn. 5, 173–194. doi: 10.1007/s11409-010-9056-2

[ref47] Lai M. H. C. Yoon M. (2015). A modified comparative fit index for factorial invariance studies. Struct. Equ. Model. Multidiscip. J. 22, 236–248. doi: 10.1080/10705511.2014.935928

[ref48] Lee Y. Cho E. Roseth C. J. (2020). Interpersonal predictors and outcomes of motivational profiles in middle school. Learn. Individ. Differ. 81:101905. doi: 10.1016/j.lindif.2020.101905

[ref49] Lent R. W. Brown S. D. (2019). Social cognitive career theory at 25: empirical status of the interest, choice, and performance models. J. Vocat. Behav. 115:103316. doi: 10.1016/j.jvb.2019.06.004

[ref50] Li C.-H. (2016). Confirmatory factor analysis with ordinal data: comparing robust maximum likelihood and diagonally weighted least squares. Behav. Res. Methods 48, 936–949. doi: 10.3758/s13428-015-0619-7, 26174714

[ref51] Li T. Wang Z. (2022). Disaggregating the between-person and within-person associations between peer acceptance and academic achievement in early elementary school. J. Appl. Dev. Psychol. 78:101357. doi: 10.1016/j.appdev.2021.101357, 34840378 PMC8623689

[ref52] Li J. Zhang Y. Gao L. Qiu Z. Zhou Y. Guo Y. . (2025). Construction and initial validations of the major decision-making self-efficacy scale (MDMSES) for Chinese high school students. BMC Psychol. 13:81. doi: 10.1186/s40359-025-02405-9, 39876007 PMC11773702

[ref53] Lin X. Lei P. Tan Q. Xiong B. (2025). Culture, goal orientation and achievement of vocational college students. Front. Psychol. 16:1639938. doi: 10.3389/fpsyg.2025.1639938, 40918287 PMC12412221

[ref54] Little T. Dawson P. Boud D. Tai J. (2024). Can students’ feedback literacy be improved? A scoping review of interventions. Assess. Eval. High. Educ. 49, 39–52. doi: 10.1080/02602938.2023.2177613

[ref55] Magee M. Kuijpers M. Runhaar P. (2022). How vocational education teachers and managers make sense of career guidance. Br. J. Guid. Couns. 50, 273–289. doi: 10.1080/03069885.2021.1948970

[ref56] McGeough R. Rudick C. K. (2018). “It was at the library; therefore it must be credible”: mapping patterns of undergraduate heuristic decision-making. Commun. Educ. 67, 165–184. doi: 10.1080/03634523.2017.1409899

[ref57] Mercer N. Howe C. (2012). Explaining the dialogic processes of teaching and learning: the value and potential of sociocultural theory. Learn. Cult. Soc. Interact. 1, 12–21. doi: 10.1016/j.lcsi.2012.03.001

[ref58] Mulder J. D. Hamaker E. L. (2021). Three extensions of the random intercept cross-lagged panel model. Struct. Equ. Model. Multidiscip. J. 28, 638–648. doi: 10.1080/10705511.2020.1784738

[ref59] Nieminen J. H. Carless D. (2023). Feedback literacy: a critical review of an emerging concept. High. Educ. 85, 1381–1400. doi: 10.1007/s10734-022-00895-9

[ref60] Niu C. Jiaxin D. Liu Y. (2025). Impact of physical exercise habit on career decision-making behavior in college students: a chain mediating effect of self-efficacy and psychological resilience. Front. Psychol. 16:1647860. doi: 10.3389/fpsyg.2025.1647860, 40904405 PMC12401895

[ref61] Obrien R. M. (2007). A caution regarding rules of thumb for variance inflation factors. Qual. Quant. 41, 673–690. doi: 10.1007/s11135-006-9018-6

[ref62] Patchan M. M. Rambo-Hernandez K. E. Deitz B. N. McNeill J. (2022). Using peer assessment to improve middle school mathematical communication. J. Educ. Res. 115, 146–160. doi: 10.1080/00220671.2022.2074948

[ref63] Pitt E. Norton L. (2017). Now that’s the feedback I want!’ Students’ reactions to feedback on graded work and what they do with it. Assess. Eval. High. Educ. 42, 499–516. doi: 10.1080/02602938.2016.1142500

[ref64] Podsakoff P. M. MacKenzie S. B. Lee J.-Y. Podsakoff N. P. (2003). Common method biases in behavioral research: a critical review of the literature and recommended remedies. J. Appl. Psychol. 88, 879–903. doi: 10.1037/0021-9010.88.5.879, 14516251

[ref65] Rakoczy K. Pinger P. Hochweber J. Klieme E. Schütze B. Besser M. (2019). Formative assessment in mathematics: mediated by feedback’s perceived usefulness and students’ self-efficacy. Learn. Instr. 60, 154–165. doi: 10.1016/j.learninstruc.2018.01.004

[ref66] Raković M. Bernacki M. L. Greene J. A. Plumley R. D. Hogan K. A. Gates K. M. . (2022). Examining the critical role of evaluation and adaptation in self-regulated learning. Contemp. Educ. Psychol. 68:102027. doi: 10.1016/j.cedpsych.2021.102027

[ref67] Raynham H. Cooper M. Hayes J. Rae J. Pearce P. (2023). Helpful and unhelpful factors in school-based counselling and pastoral care as usual: analysis of qualitative data from the experience of service questionnaire. Emotional. Behav. Difficulties 28, 234–248. doi: 10.1080/13632752.2023.2276022

[ref68] Raynham H. Jinks G. (2022). Do teaching staff in primary schools perceive any impacts of school-based counselling on school engagement? Br. J. Guid. Couns. 50, 230–247. doi: 10.1080/03069885.2021.1904502

[ref69] Ren J. Guo J. Li H. (2025). Linking digital competence, self-efficacy, and digital stress to perceived interactivity in AI-supported learning contexts. Sci. Rep. 15:33182. doi: 10.1038/s41598-025-18873-3, 41006796 PMC12475259

[ref70] Riley W. T. Martin C. A. Rivera D. E. Hekler E. B. Adams M. A. Buman M. P. . (2015). Development of a dynamic computational model of social cognitive theory. Transl. Behav. Med. 6, 483–495. doi: 10.1007/s13142-015-0356-6, 27848208 PMC5110484

[ref71] Russell J. M. Baik C. Ryan A. T. Molloy E. (2022). Fostering self-regulated learning in higher education: making self-regulation visible. Act. Learn. High. Educ. 23, 97–113. doi: 10.1177/1469787420982378

[ref72] Ryu E. (2011). Effects of skewness and kurtosis on normal-theory based maximum likelihood test statistic in multilevel structural equation modeling. Behav. Res. Methods 43, 1066–1074. doi: 10.3758/s13428-011-0115-7, 21671139

[ref73] Schweder S. Hagenauer G. Grahl L. Raufelder D. (2025). Achievement goal profiles and profile transitions in teacher- and self-directed learning and the association with self-efficacy and interest. Motiv. Emot. 49, 593–623. doi: 10.1007/s11031-025-10142-0

[ref74] Seah R. Horne M. (2020). The construction and validation of a geometric reasoning test item to support the development of learning progression. Math. Educ. Res. J. 32, 607–628. doi: 10.1007/s13394-019-00273-2

[ref75] Smit R. Dober H. Hess K. Bachmann P. Birri T. (2023a). Supporting primary students’ mathematical reasoning practice: the effects of formative feedback and the mediating role of self-efficacy. Res. Math. Educ. 25, 277–300. doi: 10.1080/14794802.2022.2062780

[ref76] Smit R. Hess K. Taras A. Bachmann P. Dober H. (2023b). The role of interactive dialogue in students’ learning of mathematical reasoning: a quantitative multi-method analysis of feedback episodes. Learn. Instr. 86:101777. doi: 10.1016/j.learninstruc.2023.101777

[ref77] Stephen J. S. Rockinson-Szapkiw A. J. Dubay C. (2020). Persistence model of non-traditional online learners: self-efficacy, self-regulation, and self-direction. Am. J. Distance Educ. 34, 306–321. doi: 10.1080/08923647.2020.1745619

[ref78] Taylor R. D. Oberle E. Durlak J. A. Weissberg R. P. (2017). Promoting positive youth development through school-based social and emotional learning interventions: a meta-analysis of follow-up effects. Child Dev. 88, 1156–1171. doi: 10.1111/cdev.12864, 28685826

[ref79] Tibbe T. D. Montoya A. K. (2022). Correcting the bias correction for the bootstrap confidence interval in mediation analysis. Front. Psychol. 13:810258. doi: 10.3389/fpsyg.2022.810258, 35712166 PMC9197131

[ref80] Wang D. Liu X. Deng H. (2022). The perspectives of social cognitive career theory approach in current times. Front. Psychol. 13:1023994. doi: 10.3389/fpsyg.2022.1023994, 36533045 PMC9749854

[ref81] Webb N. M. Franke M. L. Ing M. Turrou A. C. Johnson N. C. Zimmerman J. (2019). Teacher practices that promote productive dialogue and learning in mathematics classrooms. Int. J. Educ. Res. 97, 176–186. doi: 10.1016/j.ijer.2017.07.009

[ref82] Wei W. Cheong C. M. Zhu X. Lu Q. (2024). Comparing self-reflection and peer feedback practices in an academic writing task: a student self-efficacy perspective. Teach. High. Educ. 29, 896–912. doi: 10.1080/13562517.2022.2042242

[ref83] Williams L. J. McGonagle A. K. (2016). Four research designs and a comprehensive analysis strategy for investigating common method variance with self-report measures using latent variables. J. Bus. Psychol. 31, 339–359. doi: 10.1007/s10869-015-9422-9

[ref84] Winstone N. E. Nash R. A. Parker M. Rowntree J. (2017). Supporting learners’ agentic engagement with feedback: a systematic review and a taxonomy of recipience processes. Educ. Psychol. 52, 17–37. doi: 10.1080/00461520.2016.1207538

[ref85] Xiong Q. Fang X. Wu Y. Chen H. Hu W. Zhang Y. (2023). Guidance and counseling relations to high school students’ positive development and psychopathology: a non-recursive modeling study. Curr. Psychol. 42, 4609–4619. doi: 10.1007/s12144-021-01722-7

[ref86] Xu T. Wu X. Sun S. Kong Q. (2023). Cognitive diagnostic analysis of students’ mathematical competency based on the DINA model. Psychol. Sch. 60, 3135–3150. doi: 10.1002/pits.22916

[ref87] Xu W. Zhang L. Hu X. Zhou D. (2021). Impacts of after-action reviews on mathematical learning performance. Learn. Motiv. 76:101765. doi: 10.1016/j.lmot.2021.101765

[ref88] Xu C. Zhu P. (2024). The development and initial validation of the student perceptions of school guidance support scale: data from China. Asia Pac. Educ. Res. 33, 727–740. doi: 10.1007/s40299-023-00770-w

[ref89] Yang Y.-Y. Delgado M. R. (2025). The integration of self-efficacy and response-efficacy in decision making. Sci. Rep. 15:1789. doi: 10.1038/s41598-025-85577-z, 39805993 PMC11729858

[ref90] Yu S. Liu C. (2021). Improving student feedback literacy in academic writing: an evidence-based framework. Assess. Writing 48:100525. doi: 10.1016/j.asw.2021.100525

[ref91] Zhang Y. (2025). The impact of teacher academic support and L2 writing self on feedback-seeking behavior. Read. Writ. 38, 1197–1215. doi: 10.1007/s11145-024-10557-0

[ref92] Zhang Y. Jin Y. Li N. Li B. Chen G. (2025). Using cognitive diagnosis to guide workshop design: a preliminary study of examining novice mathematics teachers’ knowledge of dialogic teaching. Teach. Teach. Educ. 162:105059. doi: 10.1016/j.tate.2025.105059

[ref93] Zhang X. Savalei V. (2020). Examining the effect of missing data on RMSEA and CFI under normal theory full-information maximum likelihood. Struct. Equ. Model. 27, 219–239. doi: 10.1080/10705511.2019.1642111

[ref94] Zhang G. Yue X. Ye Y. Peng M. Y.-P. (2021). Understanding the impact of the psychological cognitive process on student learning satisfaction: combination of the social cognitive career theory and SOR model. Front. Psychol. 12:712323. doi: 10.3389/fpsyg.2021.712323, 34512469 PMC8427433

[ref95] Zhong J. Wen J. Li K. (2023). Do achievement goals differently orient students’ academic engagement through learning strategy and academic self-efficacy and vary by grade. Psychol. Res. Behav. Manag. 16, 4779–4797. doi: 10.2147/PRBM.S424593, 38035203 PMC10683660

[ref96] Zhu J. Yang Y. Yan Z. (2025). Psychometric properties of the feedback literacy behaviour scale (FLBS) for Chinese students. Asia Pacific J. Educ., 1–30. doi: 10.1080/02188791.2025.2537390, 41307611

[ref97] Zivin J. G. Song Y. Tang Q. Zhang P. (2020). Temperature and high-stakes cognitive performance: evidence from the national college entrance examination in China. J. Environ. Econ. Manag. 104:102365. doi: 10.1016/j.jeem.2020.102365

